# Evaluating the efficacy and safety of melatonin for sleep onset insomnia in children aged 6–12: a systematic review and meta-analysis

**DOI:** 10.1186/s12887-026-07505-y

**Published:** 2026-08-15

**Authors:** Shaimaa Alsulami, Fatima Alghurab, Rawan Alharbi, Rena Alhowaiti, Reem Alshammari, Renad Althebaiti, Dina Alharbi, Reef Albedah, Maram Alhumaidi, Atheer Alamri, Afnan Almutairi, Bashayer Alkhathlan, Lamyaa Aljohani

**Affiliations:** 1 Pharmaceutical Practices Department, College of Pharmacy, Umm Al-Qura University, Makkah, Saudi Arabia; 2 College of Pharmacy, Imam Abdulrahman Bin Faisal University, Dammam, Saudi Arabia; 3 Maternity and Children Hospital, Qassim, Saudi Arabia; 4 College of Pharmacy, Princess Nourah Bint Abdulrahman University, Riyadh, Saudi Arabia; 5 College of Medicine, Imam Abdulrahman Bin Faisal University, Dammam, Saudi Arabia; 6 College of Pharmacy, Umm Al-Qura University, Makkah, Saudi Arabia; 7 College of Pharmacy, Qassim University, Qassim, Saudi Arabia; 8 College of Pharmacy, AlMaarefa University, Riyadh, Saudi Arabia; 9 Armed Forces Hospital, Taif, Saudi Arabia; 10 College of Pharmacy, Taibah University, Madinah, Saudi Arabia; 11 Collage of Medicine, Tabuk University, Tabuk, Saudi Arabia; 12 College of Pharmacy, King Saud University, Riyadh, Saudi Arabia

**Keywords:** Melatonin, Sleep onset insomnia, Sleep onset time, Total sleep time, Safety, Pediatric

## Abstract

**Background:**

Melatonin is an endogenous hormone that regulates the body’s sleep-wake cycles. Due to several clinical trials, it is currently used as an exogenous supplement in pediatric patients who suffer from sleep-onset insomnia (SOI). Consequently, this systematic review and meta-analysis aimed to assess the evidence for the clinical efficacy of melatonin for SOI in children aged 6–12.

**Methods:**

This systematic review and meta-analysis evaluated the efficacy and safety of Melatonin for sleep-onset insomnia in children aged 6–12 by conducting literature searches in PubMed (97), Google Scholar (111), OVID (33), and Web of Science (156) databases. We performed statistical tests using RevMan for meta-analysis. Heterogeneity was evaluated using the I2 statistic. To maintain the validity of the studies, each included study was assessed by the Cochrane Collaboration Risk of Bias (ROB) tool. The outcomes investigated were sleep onset time (SOT), sleep onset latency (SOL), total sleep time (TST), all-cause dropout rates, and adverse-event dropout rates.

**Results:**

Three hundred ninety-eight records were identified, and 10 randomized clinical trials met the inclusion criteria. The results demonstrated that melatonin treatment group was associated with an earlier sleep onset time compared with placebo with a mean difference (MD) of -0.48 h (approximately 29 min earlier) (95% confidence interval [CI]: -0.93 to -0.02 h, *p* = 0.04). The heterogeneity between studies was moderate (I2 = 63%). On the other hand, it was noted that there has been noticed there was a remarkable reduction in sleep onset latency with melatonin compared to placebo, with an MD of -0.38 (95% CI: -0.57 to -0.20, *p* < 0.0001), with high heterogeneity (I2 = 82%).

**Conclusion:**

Our study confirmed that melatonin has a marked effect regarding Sleep-onset insomnia in childhood. Therefore, we found there was a statistical and clinical improvement in sleep parameters such as sleep onset time (SOT) and total sleep time (TST) which demonstrated the safety and efficacy of melatonin. However, long-term efficacy and safety need to be addressed, especially in the pediatric population.

**Supplementary Information:**

The online version contains supplementary material available at https://doi.org/10.1186/s12887-026-07505-y.

## Introduction

Sleep-onset insomnia is a prevalent concern in school-aged children, occurring in about 10–25% of this population which is associated with significant consequences for learning efficiency, emotional regulation, and overall health [1]. Chronic sleep disturbances in this age group are associated with a higher risk for academic underachievement, behavioral difficulties and mental health disorders, including anxiety and depression. Given that behavioral interventions may not always work or could be impractical, attention has turned toward pharmacologic agents. While behavioral interventions such as sleep hygiene, or sleep restriction therapy, remain the gold standard, first-line treatment for insomnia, melatonin, a hormone of the pineal gland involved in circadian rhythm regulation, has increased in popularity as a mild and safe agent to induce sleep in children [2]. In many countries, its over-the-counter status and favorable side effect profile have helped to increase its popularity. However, this widespread availability of melatonin and its use as an over-the-counter medication presents concerning clinical challenges. Dietary supplements such as melatonin are loosely regulated, because of that, recent chemical analysis of commercial pediatric melatonin products has found that many of these products are mislabeled, as the actual content of melatonin vary wildly form what is stated on the label. Moreover, it has been revealed that some products contained additional unapproved ingredients that are not listed on the label, such as cannabidiol (CBD), which presents a considerable risk of toxicity, and it highlights the importance of clinical monitoring and parental education [3].

Recent data indicate that short-term courses of melatonin are efficacious in improving sleep latency in pediatric patients with idiopathic chronic sleep-onset insomnia (ICSOI) and delayed sleep-wake phase disorder (DSWPD) [4]. Initial RCTs have shown that melatonin use decreased sleep onset and improved total sleep time and quality of life in insomnia, with a small effect size [2, 4]. While melatonin is most effective when aligned with the circadian phase, it requires careful monitoring by the parents. The existing evidence is of moderate quality, and there is a gap in the literature regarding the long-term safety for pediatrics. 

There has been a minimal rate of dropout due to adverse effects, and such trials have also demonstrated acceptance to both parents and children. Melatonin's potential for treating children’s sleep disturbances is further supported by evidence showing that it enhances both morning alertness and overall sleep quality [1, 2].

However, although now widely used in clinical practice, the optimal dosing, duration of treatment, long-term safety, and the size of effect in different pediatric subgroups remain uncertain. This is especially true for young children in whom developmental differences can affect both pharmacokinetics and response to treatment. Furthermore, there are specific concerns about melatonin effects on pubertal maturation and hormonal control that call for careful long-term follow-up. These gaps underline the importance of effectively and appropriately synthesizing the available data to enable evidence-based decision-making and guarantee optimal therapeutic use.

This systematic review and meta-analysis aim to assess the effectiveness and safety of melatonin in the treatment of sleep-onset insomnia in children aged 6-12 years old through the analysis of randomized controlled trials and high-quality observational studies. Considering recent findings and reducing methodological variability, this meta-analysis aims to contribute to clinical knowledge and guide new treatment approaches. It is also attempting to elucidate factors that will determine appropriate treatment selection and subgroups for whom the treatment will be most effective, thus refining sleep disorder management of children.

## Methodology

### Literature search

This systematic review was registered in the International Prospective Register of Systematic Reviews (PROSPERO) (registration number: CRD42024581245). A thorough review of the literature was conducted from August 25 to September 9, 2024, with the aim to identify relevant studies evaluating the safety and effectiveness of melatonin for children with sleep-onset insomnia aged from 6 to 12 years. OVID, Web of Science, Google Scholar, and PubMed were among the databases we consulted to make sure we had a thorough evaluation of the literature. Although no date restrictions were applied to the search, all included studies were published between 2001 and 2018. Among the terms we included in our search strategy were melatonin, children, sleep-onset insomnia, and chronic sleep disorders. The PICO framework was used to establish our search strategy: population (children aged 6–12 with sleep-onset insomnia), intervention (melatonin), comparison (no intervention or placebo), and outcome. Primary outcomes were defined as sleep onset time (SOT) and sleep onset latency (SOL). Secondary outcomes included total sleep time (TST), dim light melatonin onset (DLMO), and dropout rates (due to all causes and adverse events). To ensure consistency in data extraction, the following definitions were applied: Sleep Onset Time (SOT) refers to the specific clock time at which the participant falls asleep, Sleep Onset Latency (SOL) is the duration of time in minutes from the point of “lights-off” until the actual onset of sleep, Total Sleep Time (TST) stands for the total duration of sleep throughout the night, and Dim Light Melatonin Onset (DLMO is the timing of the endogenous increase in melatonin secretion under dim light conditions.

This systematic review complies with the Preferred Reporting Items for Systematic Reviews and Meta-analyses (PRISMA) 2020 standards.

### Study selection

We included studies based on predetermined inclusion and exclusion criteria. The inclusion criteria included studies that used randomized controlled trials to examine the use of melatonin for pediatric patients with sleep-onset insomnia. To ensure standardized diagnostic rigor, eligible trials were required to have utilized clinical screening for bedtime insomnia based on comprehensive patient history and validated questionnaires evaluating key elements such as difficulty falling asleep, sleep latency > 30 min, wake time after sleep onset, total sleep time, and sleep efficiency. Included literature focused on identifying childhood behavioral insomnia (both sleep-onset association and limit-setting types) using assessments dependent on parental reports, sleep diaries, or actigraphy to track behavioral patterns like bedtime resistance, prolonged sleep latency, frequent waking, and parental interventions. Relevant clinical outcomes and the number of individuals using melatonin were required for eligible studies. Language and publishing date were unrestricted.

The exclusion criteria encompassed studies that focused on sleep disorders other than sleep-onset insomnia or included participants who were not within the ages of 6 to 12 years, didn’t compare melatonin to a placebo, had non-randomized designs, had low methodological quality, or had a significant risk of bias. Furthermore, insomnia secondary to medical causes or underlying comorbidities was excluded, and objective assessments like polysomnography were accepted only when utilized strictly to rule out suspected primary sleep disorders, excluding sleep-disordered breathing. Although randomized controlled trials were prioritized, high-quality observational cohort studies evaluating long-term safety and efficacy outcomes were also considered eligible for inclusion. In addition, studies using concomitant medications that were changed throughout the trial period or that did not employ melatonin as the main treatment for sleep-onset insomnia were also excluded. The screening procedure allowed for the identification and removal of duplicate publications. Two independent reviewers screened titles and abstracts for eligibility, followed by a full-text evaluation of potentially relevant articles. A comprehensive search strategy can be found in Appendix (I).

### Data extraction

To ensure consistency, a standardized form was used for data extraction. Moreover, the main variables that were extracted were the study design, sample size, patient demographics, intervention details (melatonin dosage, administration time, and duration), baseline sleep onset latency (SOL), baseline total sleep time (TST), sleep onset latency (SOL) following intervention, total sleep time (TST) following intervention, light-off time, wake-up time, and reported adverse events. Further details on medical history, dosage forms, administration timing, follow-up duration, and dropout rates were obtained to provide an in-depth overview of each study’s context and conclusions.

### Statistical analysis

We used Excel for descriptive statistics and RevMan for the meta-analysis, which included forest plots and an I² heterogeneity assessment. For categorical variables such as study design, country, dose form, and administration time, percentages and frequencies were computed. Continuous variables such as age, follow-up duration, melatonin dosage, sleep onset latency (SOL), total sleep time (TST), insomnia severity index scores, and dim light melatonin onset (DLMO) were summarized utilizing means and standard deviations (SD) for normally distributed data. Medians and interquartile ranges (IQR) were used for non-normally distributed data. For the primary outcomes (SOT and SOL) and secondary outcomes (TST, DLMO, and dropout rates), effect sizes were calculated, and 95% confidence intervals (CIs) were presented. For binary outcomes, percentages were computed using occurrence counts. The study’s heterogeneity was assessed using the I² statistic. Publication bias was assessed using appropriate techniques. Statistical significance was determined at *p* < 0.05.

### Risk of bias assessment

The Cochrane Collaboration Risk of Bias (ROB) tool was used to evaluate the risk of bias for each included study [5]. This assessment was performed independently by two investigators. Any disagreements or discrepancies between the investigators were resolved through discussion and consensus or by consultation with a third investigator.

## Results

### Study selection and characteristics

**Fig. 1:**
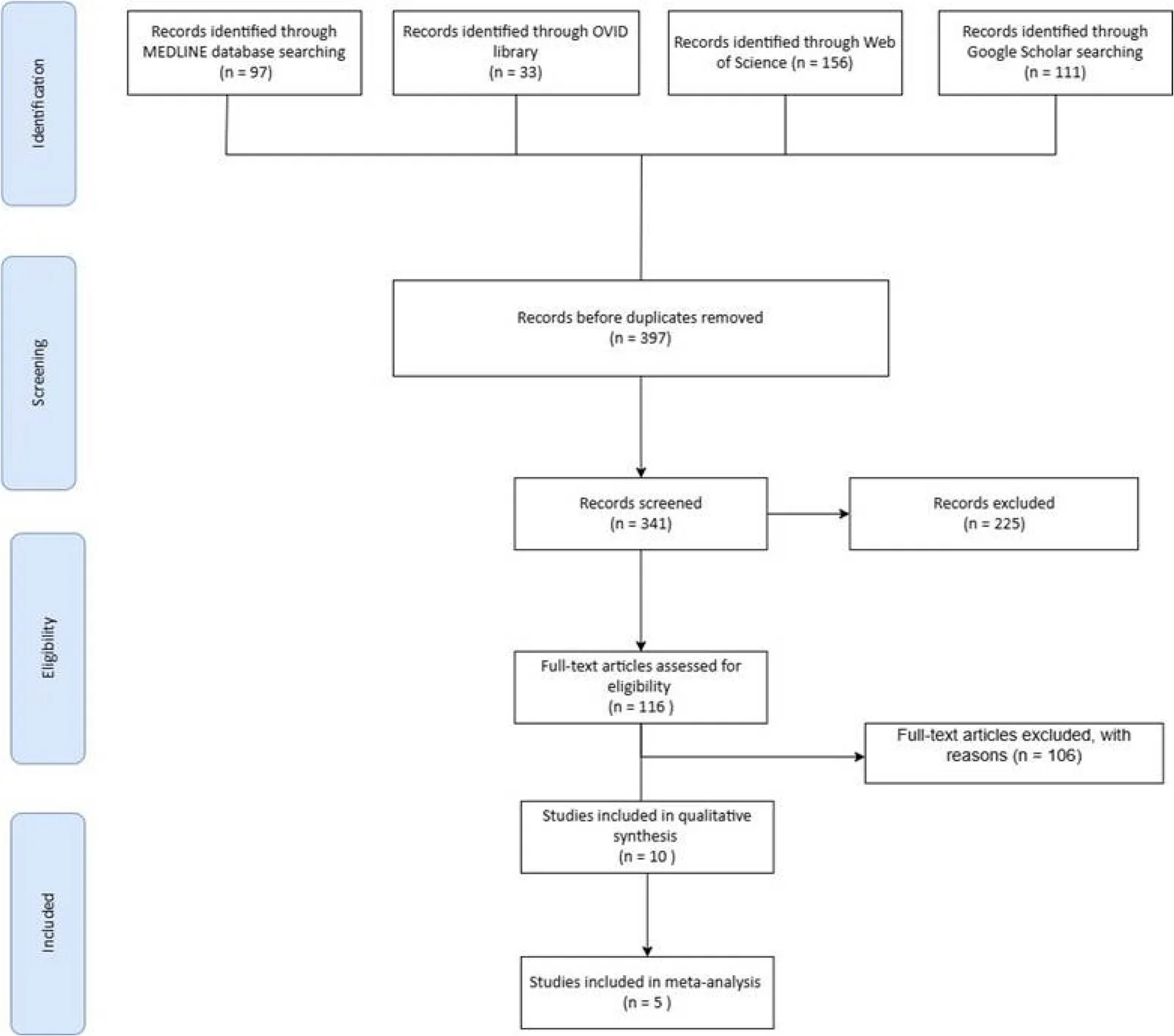
PRISMA flowchart illustrating the study selection process

The literature search was conducted across multiple databases, yielding the following results: PubMed (97), Google Scholar (111), OVID (33), and Web of Science (156), for a total of 397 records. After removing duplicates, 341 studies were eligible for screening. Titles and abstracts were independently reviewed by two assessors based on predefined inclusion and exclusion criteria, with disagreements resolved through discussion or consultation with a third reviewer. Of these, 225 studies were excluded, leaving 116 for full-text assessment. The full-text articles underwent independent review, and studies not meeting the inclusion criteria were excluded, with reasons documented. Any discrepancies were resolved by consensus or through a third reviewer. Ultimately, ten studies published between 2001 and 2018 were included in the qualitative synthesis [1, 2, 4, 6–12], and five studies were included in the meta-analysis. The PRISMA flow diagram illustrates the study selection process (Fig. 1). Study designs included 8 randomized controlled trials (RCTs) (80%) and 2 cohort studies (20%). RCTs comprised 4 double-blind, placebo-controlled trials (40%), 1 double-blind, placebo-controlled crossover study (10%), 1 placebo-controlled, double-blind clinical trial (10%), 1 double-blind study (10%), and 1 controlled study (10%). Locations included 9 studies in the Netherlands (90%) and 1 in the USA (Cincinnati) (10%) These characteristics are detailed in Table 1.

**Table 1:** Summary and characteristics of the included studies

Last Name of Author	Year	Journal	Study Design	Country	Total Number of Patients	Inclusion Criteria (Summary)	Exclusion Criteria (Summary)
Heijden	2005	J Sleep Research	Randomized, placebo-controlled, double-blind trial	Netherlands	110	Children 6–12 years with idiopathic chronic sleep onset insomnia, sleep latency > 30 min	Sleep maintenance insomnia, disturbed sleep architecture, intellectual disability, prior melatonin use, certain medications
Hoebert	2009	J Pineal Research	Randomized, double-blind study	Netherlands	105	Children 6–12 years with ADHD, IQ > 80, chronic sleep onset insomnia	Not mentioned (NM)
Jain	2015	Sleep Medicine	Randomized, double-blind crossover study	USA (Cincinnati)	11	Children 6–11 years, pre-pubertal, epilepsy, normal development, sleep issues	Obstructive sleep apnea, PLM disorder, psychiatric disorders, prior melatonin use, certain medications
Maanen	2017	Sleep Research Society (Sleep)	Randomized controlled study	Netherlands	84	Children 7–12 years with chronic sleep onset problems > 1 month, sleep latency > 30 min	Psychiatric disorders (except ADHD/autism), chronic pain, epilepsy, prior melatonin use, certain medications
Smits	2001	J Child Neurology	Randomized, double-blind, placebo-controlled trial	Netherlands	40	Children 6–12 years with chronic sleep onset insomnia > 1 year, sleep latency > 30 min	Disturbed sleep architecture, sleep maintenance insomnia, intellectual disability, prior melatonin use, certain medications
Smits	2003	J Am Acad Child Adolesc Psychiatry	Randomized, double-blind, placebo-controlled trial	Netherlands	62	Children 6–12 years, good health, idiopathic chronic sleep-onset insomnia > 1 year	Disturbed sleep architecture, sleep maintenance insomnia, intellectual disability, prior melatonin use, certain medications
Van Geijlswijk	2010	Springer	Randomized, placebo-controlled, double-blind trial	Netherlands	72	Children 6–12 years with chronic sleep onset insomnia > 1 year, sleep latency > 30 min	Psychiatric/pedagogic SOI, intellectual disability, epilepsy, chronic pain, prior melatonin use, certain medications
Van Geijlswijk	2011	Springer	Cohort study	Netherlands	59	Children 6–12 years with chronic idiopathic sleep onset insomnia > 1 year, good health	Psychiatric/pedagogic SOI, intellectual disability, epilepsy, chronic pain, prior melatonin use, certain medications
Heijden	2007	J Am Acad Child Adolesc Psychiatry	Randomized, double-blind, placebo-controlled study	Netherlands	107*	Children 6–12 years with ADHD and chronic sleep-onset insomnia, IQ > 80	Total IQ < 80, pervasive developmental disorder, epilepsy, chronic pain, prior melatonin use, certain medications
Zwart	2018	Healthcare	Cohort study	Netherlands	69	Children 6–12 years with chronic sleep onset insomnia > 1 year, non-responsive to sleep hygiene	Psychiatric/pedagogic SOI, intellectual disability, epilepsy, chronic pain, prior melatonin use, certain medications

For Heijden [9], 107 patients were enrolled, with 105 completing the study

### Patient demographics

**Fig. 2:**
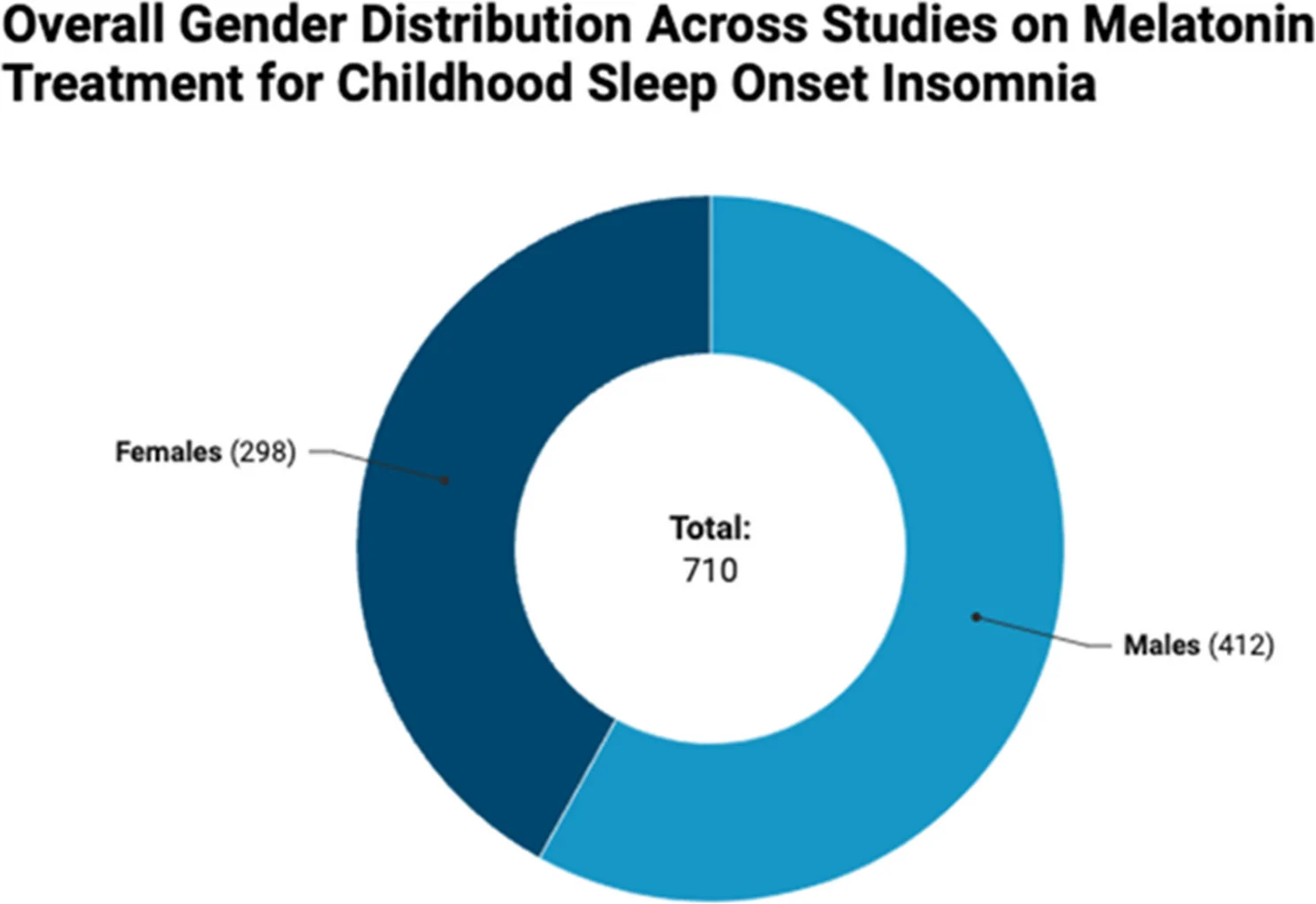
Gender distribution across the included studies

The total number of patients across the included studies was 717. Across the included studies, participants were initially enrolled between 6 and 12 years of age according to the eligibility criteria. However, in long-term follow-up cohort studies, particularly the study by Zwart et al., some participants reached older ages (up to 23.2 years) during extended follow-up assessments. The overall mean age across the studies was 0.27 years, with a pooled standard deviation (SD) of ± 1.24 years. Of the 717 participants, 412 were male (53.3%), 298 were female (42.0%) (Fig. 2). All 10 studies (100%) reported chronic sleep onset insomnia (CSOI or SOI) lasting over 1 year, with difficulty falling asleep more than 4 nights per week in 8 studies (80%). Additional medical conditions included epilepsy in 11 patients (1.5%), ADHD in 3 studies (30%) with 11 boys using methylphenidate (5 in the melatonin group, 6 in the placebo group), asthma in 2 studies (20%) with 4 children using salbutamol, and 1 child using lactitol for chronic constipation. Co-medications reported in 2 studies (20%) included antihistamines (desloratadine, ketotifen, levocetirizine, hydroxyzine), fluticasone, valproic acid, trimethoprim, and lactitol. One study (10%) noted disruptive behavior disorders and anxiety disorders.

Several included studies involved participants with comorbid conditions such as ADHD, epilepsy, and asthma, as well as the use of concomitant medications. However, subgroup analyses according to comorbidities or concomitant medication use were not feasible because of limited sample sizes and insufficiently stratified data across the included studies.

### Melatonin treatment characteristics

Melatonin dosages across 9 studies ranged from 0.3 mg to 10 mg, with an overall mean dose of 4.06 mg; 1 study (10%) used weight-based doses of 0.05 mg/kg, 0.1 mg/kg, and 0.15 mg/kg. The specific treatment parameters are summarized in Table 2. Dosage forms included sustained release melatonin in 1 study (10%), capsules in 1 study (10%), fast-release tablets in 2 studies (20%), fast-release tablet mixed with carboxymethylcellulose in 1 study (10%), tablets in 1 study (10%), and were not specified in 4 studies (40%). The administration time for melatonin across studies ranged from 18:00 to 23:00, with a mean administration time of approximately 19:29 (± 48 min). Most studies recommended administering melatonin 30 min to 1 h before bedtime. The duration of treatment across the studies varied widely, ranging from 3 weeks to approximately 11.9 years, with a mean duration of 100.25 weeks (± 181.07 weeks). The mean duration of treatment across studies is 76.73 weeks, ± 153.31 weeks. The treatment duration ranges from 0.86 weeks (6 nights) to 516 weeks (9.6 to 11.9 years).

**Table 2:** Melatonin treatment details across studies

Last Name of Author	Melatonin Dosage	Dosage Form	Administration Time	Duration of Treatment
Heijden	5 mg	Not mentioned (NM)	18:00 or 19:00 h	4 weeks
Heijden	3 mg (< 40 kg); 6 mg (> 40 kg)	Fast-release tablets	7:00 PM	4 weeks
Hoebert	0.5–10 mg (mean: 4.23 ± 0.27 mg)	Not mentioned (NM)	18:30–23:00 (mean: 19:53)	1–57 months (mean: 18.28 ± 3.0)
Jain	9 mg	Sustained-release melatonin	30 min prior to bedtime	4 weeks
Maanen	3 mg	Tablets	19:00 h	3–4 weeks
Smits	5 mg	Fast-release tablet	6:00 PM	4 weeks + 1-week washout + 4-week crossover
Smits	5 mg	Fast-release tablet mixed with carboxymethylcellulose	7:00 PM	4 weeks
Van Geijlswijk	0.05–0.15 mg/kg (by group)	Capsules	17:58–20:42 h	6 nights
Van Geijlswijk	Mean: 2.7 mg (0.3–10 mg range)	Not applicable (N/A)	Not applicable (N/A)	3.1 years
Zwart	0.5–5.0 mg	Not mentioned (NM)	19:00–23:00	9.6–11.9 years

#### Clinical outcomes and meta-analysis

**Fig. 3:**
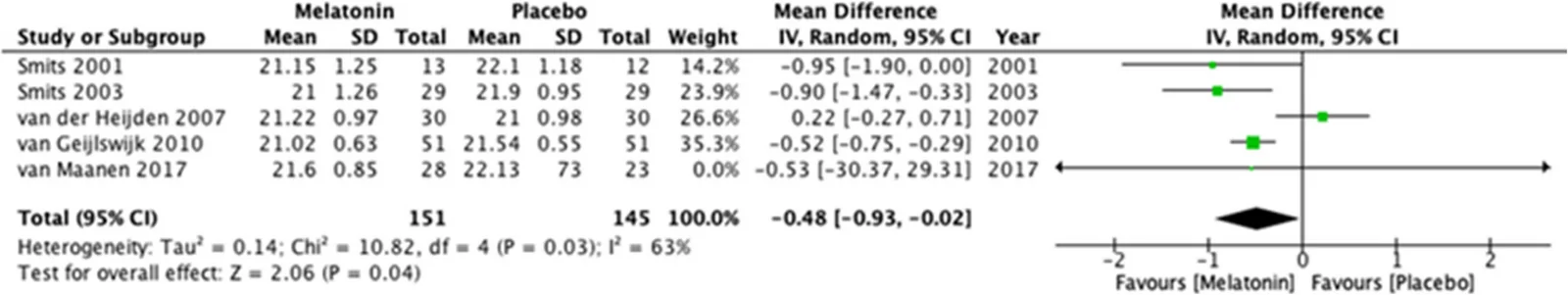
Forest plot of mean difference in sleep onset time with melatonin vs. placebo

**Fig. 4:**
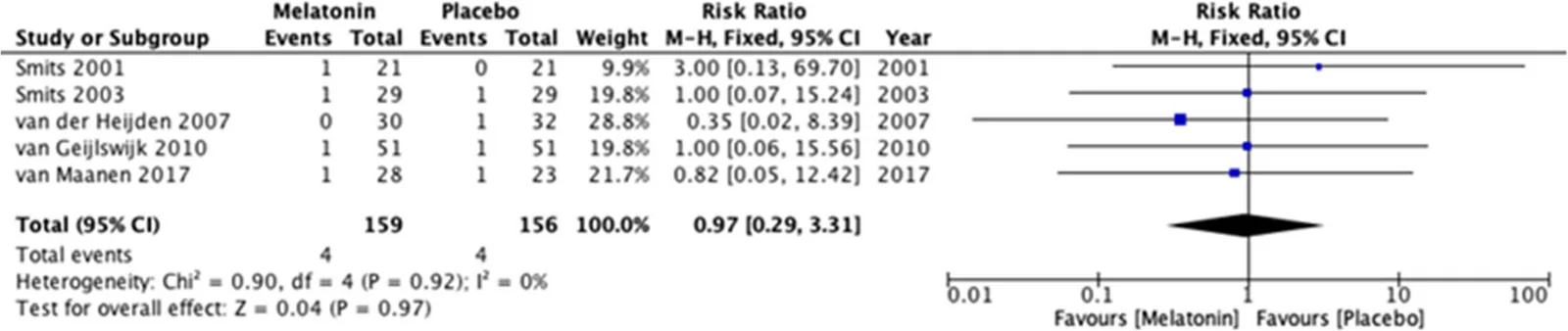
Forest plot of risk ratio for dropout rates with melatonin vs. placebo

**Fig. 5:**
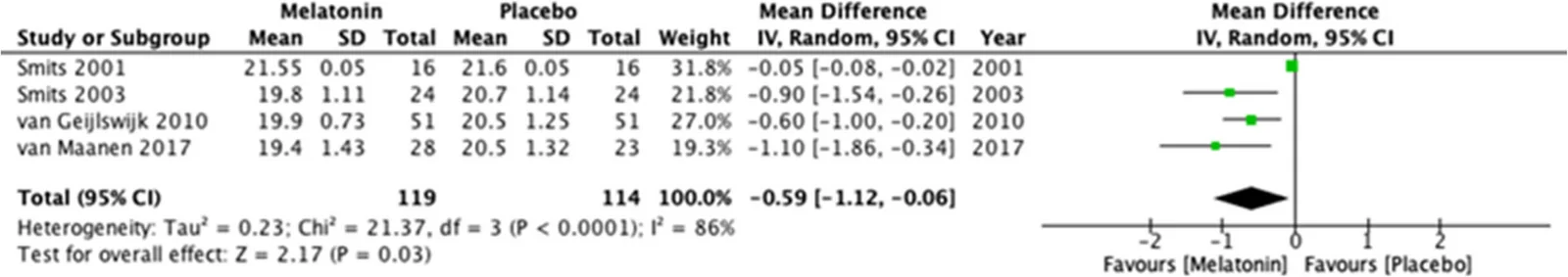
Forest plot of mean difference in Dim Light Melatonin Onset (DLMO) with melatonin vs. placebo

**Fig. 6:**
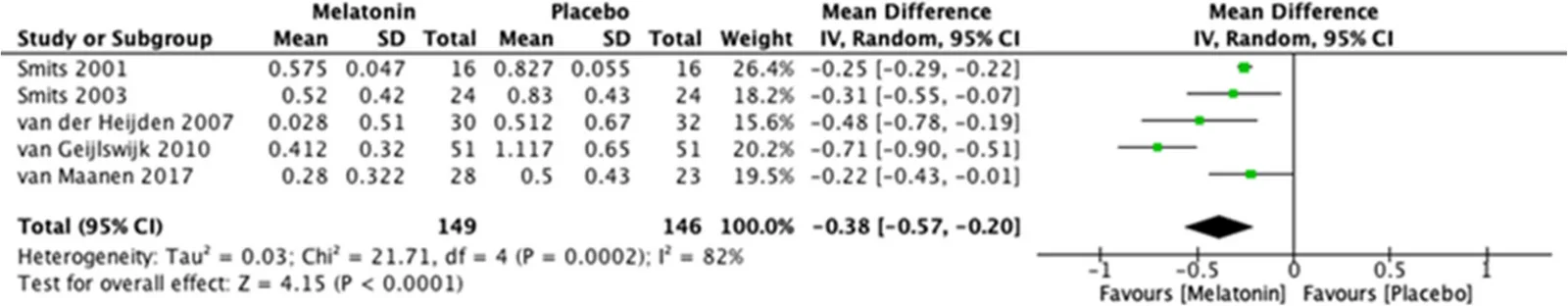
Forest plot of mean difference in Sleep Onset Latency (SOL) with melatonin vs. placebo

**Fig. 7:**
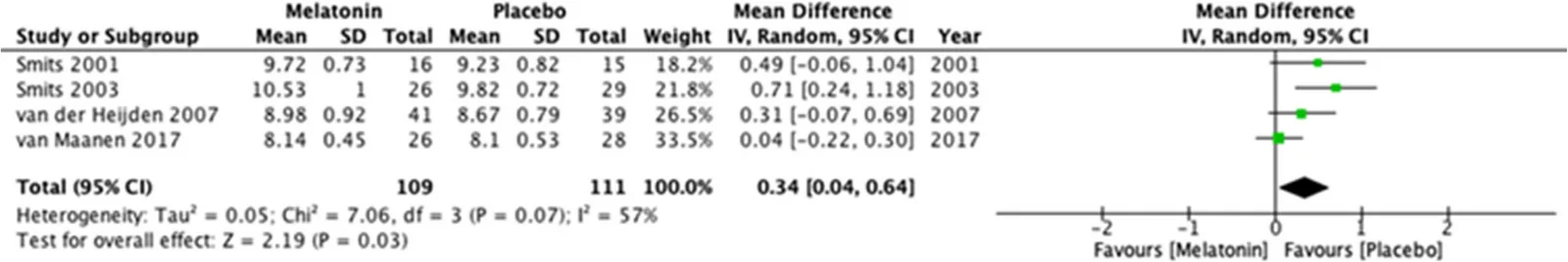
Forest plot of mean difference in Total Sleep Time (TST) with melatonin vs. placebo

Melatonin treatment was associated with an earlier sleep onset time compared with placebo. Meta-analysis demonstrated a mean difference of -0.48 h (approximately 29 min earlier sleep onset) (95% CI: -0.93 to -0.02 h, *P* = 0.04). The meta-analysis evaluated several sleep-related outcomes and dropout rates to assess the efficacy and tolerability of melatonin compared to placebo. Detailed findings, limitations, research gaps, and recommendations from these studies are presented in Table 3. It revealed a significant reduction in sleep onset time with melatonin compared to placebo, with a mean difference (MD) of -0.48 h (95% confidence interval [CI]: -0.93 to -0.02, *P* = 0.04) (Fig. 3), although heterogeneity among the studies was moderate, with an I² value of 63%. Additionally, a significant reduction in sleep onset latency was observed with melatonin compared to placebo, with an MD of -0.38 (95% CI: -0.57 to -0.20, *P* < 0.0001) (Fig. 6), but heterogeneity was high, with an I² value of 82%. The meta-analysis also demonstrated a significant increase in total sleep time with melatonin compared to placebo, with an MD of 0.34 (95% CI: 0.04 to 0.64, *P* = 0.03) (Fig. 7), with moderate heterogeneity (I² = 57%). Furthermore, a significant advancement in dim light melatonin onset was noted with melatonin compared to placebo, with an MD of -0.59 (95% CI: -1.12 to -0.06, *P* = 0.03) (Fig. 5), though heterogeneity was high, with an I² value of 86%. Regarding tolerability, no significant difference was observed in the risk of dropout for any cause between the melatonin and placebo groups, with a risk ratio (RR) of 0.97 (95% CI: 0.29 to 3.31, *P* = 0.97) (Fig. 4), and heterogeneity was low, with an I² value of 0%. However, the interpretation of these findings should be approached cautiously because several included studies had relatively small sample sizes. In particular, the study by Jain et al. included only 11 participants, which may have reduced the statistical power and stability of the pooled estimates. Additionally, the relatively small overall study population may limit the generalizability of the findings.

**Table 3:** Key findings, limitations, study gaps, and recommendations across studies on melatonin for sleep onset insomnia

Last Name of Author	Main Findings	Limitations	Study Gaps	Recommendations
Heijden	Children with idiopathic chronic SOI: Early evening melatonin treatment advanced sleep onset and endogenous onset, more effective with delayed pretreatment onset.	Actigraphy/saliva sample data loss, fixed melatonin timing ignored individual circadian variability, small sample size reduction.	Limited exploration of melatonin’s soporific vs. chronobiotic effects, lack of long-term data, no comparison with other treatments, limited generalizability.	Use melatonin cautiously after non-pharmacological treatments fail, investigate long-term effects and developmental impacts.
Heijden	Melatonin advanced sleep onset (~ 1 h) and total sleep time (41 min) in ADHD/SOI children, no effect on behavior, cognition, or quality of life, no significant adverse events.	2-year follow-up may miss long-term effects, excludes stimulant-treated ADHD children, missing behavior/quality-of-life data, actigraphy limits sleep quality assessment.	No investigation of melatonin’s effects on specific ADHD behaviors, sleep quality, or external factors; lacks long-term data on diverse populations.	Explore long-term melatonin effects on sleep, behavior, and cognition in diverse ADHD populations, including stimulant users; use polysomnography for sleep quality.
Hoebert	Melatonin effectively treated sleep onset insomnia in ADHD children long-term but didn’t cure it; discontinuation led to relapse, resuming treatment common.	Lack of systematic co-medication data, no measures for long-term pubertal/fertility effects, retrospective design, no control group.	Limited data on adverse events timing, impact of comorbidities (e.g., anxiety, depression), and pubertal/fertility effects.	Research melatonin’s impact on pubertal development, efficacy across neurodevelopmental disorders, and measure pretreatment DLMO.
Jain	Sustained-release melatonin reduced sleep latency (*p* = 0.02) and waking after sleep onset (*p* = 0.04) in epileptic children, improved N3 sleep, prolonged REM latency, no increased seizures.	Small sample size, excluded refractory seizures, data loss in two subjects affected secondary outcomes, no significant actigraphy/subjective sleep changes.	Limited understanding of melatonin’s role in refractory epilepsy, insufficient power for secondary outcomes, need for EEG/long-term effect research.	Conduct larger studies on melatonin’s efficacy, safety, and tolerance in epilepsy, including diverse epilepsy types and sleep disturbances.
Maanen	Melatonin treatment was more effective than light therapy for SOI with delayed melatonin onset in children, making it the preferred clinical option.	Fixed light therapy timing, no placebo for light therapy, uncontrolled evening light exposure, unassessed sleep hygiene impact, short duration, lack of DLMO norms.	Limited research on light therapy for pediatric SOI, optimal dosage/timing for both treatments, long-term impacts, specific groups (e.g., psychiatric disorders), sleep hygiene effects.	Research different light therapy protocols, conduct long-term studies on melatonin/light therapy effects, target specific groups, explore sleep hygiene integration, optimize dosage/timing.
Smits	Melatonin advanced sleep onset (57 min), reduced sleep latency (17 min), advanced melatonin onset (82 min) in idiopathic SOI, improved health/sleep, modest placebo effect from parental attention.	Dutch-specific population, potential ADHD bias, no objective sleep measures (e.g., actigraphy), short follow-up.	Lack of long-term melatonin effects, limited generalizability, no comparison with other treatments, unclear optimal dose.	Investigate long-term safety/efficacy in diverse populations, limit melatonin to severe resistant insomnia with delayed onset, conduct multi-center trials.
Smits	Melatonin significantly advanced sleep onset (~ 1 h) and dim light melatonin onset (57 min), increased lights-off time (34 min), total sleep time (41 min), no change in attention/wake-up time.	Small sample (40 children), short treatment (4 weeks), limited long-term safety data, uncertainty in optimal dosage/timing.	Focus on short-term effects, uncertainty in optimal dosage/timing, mechanism of action, comparison with other sleep therapies, generalizability to broader populations.	Determine optimal dosage/timing, assess long-term safety/efficacy and reproductive effects, use lowest effective dose, evaluate annually for discontinuation.
Van Geijlswijk	Melatonin (0.05 mg/kg) effectively treated CSOI, advanced sleep onset (1 h), dim light melatonin onset (1 h), reduced sleep latency (35 min), no dose-response (0.05–0.15 mg/kg).	Small sample (16–19/group), wide DLMO-TOA intervals, naturally determined TOA.	No dose-response above 0.05 mg/kg, need for long-term safety, individual bioavailability studies.	Evaluate dosage-TOA relationship with narrower DLMO-TOA, report all p-values, conduct long-term studies on adverse events and chronic use.
Van Geijlswijk	Melatonin use persisted in 81% of children after 3.1 years, 6/11 discontinuers resolved sleep issues, no rebound effects, low adverse events (e.g., 58% headaches, normal population rate), normal social development, poorer sleep vs. controls, no pubertal changes, high user satisfaction.	Small sample (57 for long-term), single clinic, non-representative of healthy Dutch children, invalidated CSHQ for older children, short follow-up.	No dose-response analysis, Dutch-only population, limited long-term data (> 3.1 years), no comparison with other interventions, no drug interactions.	Monitor long-term melatonin safety (puberty, sleep, mental health), conduct studies beyond 3 years, tailor dosing, track side effects.
Zwart	Long-term melatonin therapy (7.1 years) was safe, 75% of CSOI children had normal sleep without medication after 10 years.	Low statistical power, recall bias, self-reported sleep timing (no actigraphy/ polysomnography), potential overestimation of melatonin continuation, student controls may not represent general population.	Need for objective sleep measures, diverse demographics, factors in therapy cessation.	Assess long-term effects on development (physical, psychological, social) into adulthood, use diverse populations, explore chronotype-daily functioning links, investigate biological/environmental factors.

### Safety and adherence

Nine studies (90%) reported adverse events. Headache was reported in six studies (*n* = 47, 6.6%), dizziness in four studies (*n* = 12), nausea in four studies (*n* = 5), and decreased mood in three studies. An additional adverse event was a decrease in appetite in one child and stomachache in another. Weight-related effects were observed in two children (weight gain) and apathy accompanying weight gain in one child. A total of seven children reported experiencing urinary symptoms, including nocturnal diuresis (*n* = 3), enhanced urination (*n* = 2), bedwetting (*n* = 1), and increased urinary frequency (*n* = 1). Agitation (*n* = 1), apathy (*n* = 1), emotive mood changes (*n* = 1), and a short temper during high-dose therapy were among the neuropsychiatric and behavioral events. Other reported side effects included excessive morning sedation (*n* = 1), visual disturbances (*n* = 1), constipation (*n* = 1),Pale look, cold sensations (*n* = 8), and red cheeks, red earlobes, red eyes and yawning (*n* = 15). Bronchitis and otitis were the two infections that were reported. Four months after commencing treatment, one child developed mild generalized epilepsy, although the causal relationship was ambiguous. In general, these adverse events were mild, transient, or dose-related, with severe events being exceedingly uncommon.

Although treatment duration ranged from 6 nights to approximately 11.9 years across the included studies, most randomized controlled trials evaluated melatonin use over relatively short periods, commonly between 3 and 4 weeks. Only a limited number of observational follow-up studies assessed long-term melatonin use beyond 2–3 years, and these studies were limited by relatively small sample sizes, retrospective or cohort designs, lack of control groups, and reliance on self-reported or subjective sleep assessments. Furthermore, long-term developmental outcomes, including pubertal maturation, endocrine function, neurodevelopment, cognitive performance, behavioral outcomes, and psychosocial functioning, were not systematically or consistently evaluated across the included studies.

### Bias assessment

**Fig. 8:**
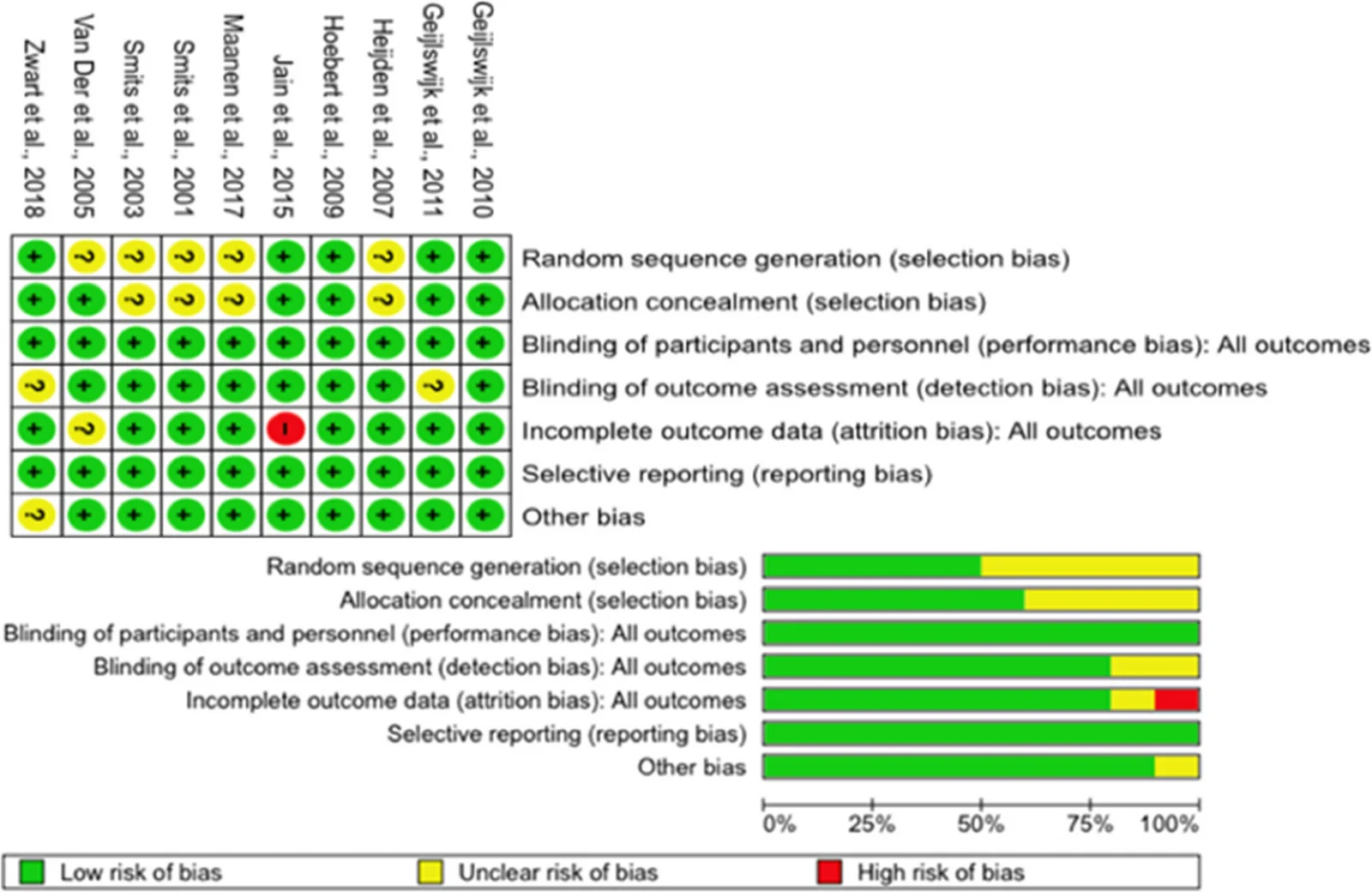
The risk of bias assessment for the included randomized controlled trials using the cochrane risk of bias tool

A total of 10 studies were included in this systematic review, all of the articles are RCTs. The risk of bias assessment for the included RCTs using the Cochrane Collaboration ROB tool revealed that all of the studies had a low risk of bias. The most common sources of bias were domain 1 and domain 4. (Fig. 8) illustrating the risk of bias across key domains for each included study.

## Discussion

Our systematic review and meta-analysis show that melatonin is both effective and well-tolerated for treating sleep onset insomnia (SOI) in children aged 6–12 years. It significantly reduces sleep onset time (SOT) and sleep onset latency (SOL), while also increasing total sleep time (TST) and advancing dim light melatonin onset (DLMO). These findings are consistent with previous research, such as the study by van der Heijden et al. [13], which found that melatonin effectively advanced sleep onset and endogenous melatonin secretion in children with idiopathic chronic SOI, particularly in those with delayed baseline melatonin onset. Likewise, Smits et al. [4] demonstrated that melatonin not only reduces sleep latency but also advances sleep onset and showed notable improvements in overall sleep patterns in children with chronic SOI. Another study by Smits et al. [2] highlighted an additional benefit of melatonin on sleep and overall well-being in children with SOI. However, it also pointed out that some improvements could be attributed to parental attention (a modest placebo effect). Overall, these findings reinforce melatonin’s role in improving sleep timing and duration in children with SOI.

It is also important to consider the role of behavioral and sleep hygiene interventions when interpreting the efficacy of melatonin in pediatric SOI. Behavioral strategies, including consistent bedtime routines, sleep hygiene education, and limitation of evening screen exposure, are generally recommended as first-line therapy and may independently improve sleep outcomes. Variability in adherence to these non-pharmacological measures across studies may therefore represent a potential confounding factor influencing the observed treatment effects attributed to melatonin. In clinical practice, melatonin appears to be most beneficial when administered according to the child’s circadian phase and may be particularly useful in children with delayed sleep-wake phase tendencies or comorbid ADHD. Consequently, melatonin should ideally be considered as an adjunctive or second-line option in children who do not adequately respond to behavioral interventions alone. Appropriate parental counseling, informed consent and clinical monitoring remain important, particularly during prolonged treatment.

Furthermore, our findings suggest a generally favorable tolerability profile of melatonin during the available follow-up periods, as demonstrated by Zwart et al. [11], who found that the majority of children continued to sleep as usual even after discontinuing melatonin, with minimal adverse effects noted. Similarly, van Geijlswijk et al. [6] found that 81% of children who had been taking melatonin for 3.1 years continued to do so without experiencing severe adverse reactions, and those who stopped did not suffer from rebound insomnia. Additionally, van Geijlswijk et al. [8] examined dose-response relationships in another trial and found that doses above 0.05 mg/kg did not confer additional benefit. This finding is consistent with our results, which suggest that melatonin effectiveness is influenced by baseline DLMO timing and individual variability, rather than simply increasing dosage.

While melatonin has shown efficacy in treating SOI in children, our findings highlight several key limitations and areas for further research. A significant concern is the variability in treatment response reported between trials, suggesting that baseline dim light melatonin onset (DLMO), comorbid conditions, and the presence of ADHD may influence melatonin’s effectiveness. This is supported by the findings of van der Heijden et al. [9], who showed that, while melatonin dramatically improved sleep parameters in children with ADHD and SOI, it had no significant effects on behavior, cognitive function, or overall quality of life. Similarly, Hoebert et al. [10] demonstrated melatonin’s efficacy as a long-term treatment for children with ADHD and SOI, although discontinuation frequently resulted in relapse. Another important limitation of this review is the limited geographical diversity of the included studies. Nine of the ten included studies were conducted in the Netherlands, while only one study originated from the United States. This geographical concentration may limit the generalizability of the findings to broader pediatric populations worldwide. Differences in ethnicity, genetic background, lifestyle patterns, environmental light exposure, sleep habits, dietary factors, healthcare systems, and clinical practices across regions may influence both the therapeutic response and safety profile of melatonin in children. Furthermore, cultural differences in sleep behaviors and parental management of sleep disturbances may also contribute to variability in treatment outcomes. Therefore, although the available evidence supports the efficacy and tolerability of melatonin for pediatric SOI, the findings should be interpreted cautiously when applying them to populations outside the studied regions. Future multi-center studies involving geographically and ethnically diverse populations are needed to improve external validity and determine whether treatment responses remain consistent across different demographic and clinical settings.

In addition to the limitations of the included studies, some methodological limitations of this systematic review should be considered. The relatively small number of included studies and variability in study characteristics and reporting limited the ability to perform subgroup and heterogeneity analyses. In addition, publication bias and certainty of evidence were not formally assessed. Nevertheless, despite these limitations, the available evidence generally supports the potential efficacy and tolerability of melatonin for pediatric sleep onset insomnia.

Concerns remain regarding the long-term effects of melatonin on puberty, neurodevelopment, cognitive function, and endocrine regulation in pediatric populations. Smits et al. [2] and Hoebert et al. [10] stressed the importance of prolonged follow-up studies to evaluate potential developmental implications of long-term melatonin therapy. Existing long-term follow-up studies provide somewhat reassuring findings; however, the available evidence remains limited. Van Geijlswijk et al. [6] reported that children receiving long-term melatonin therapy for chronic SOI did not demonstrate significant abnormalities in pubertal development, mental health, or sleep quality compared with population norms over a mean follow-up period of 3.1 years. Similarly, Zwart et al. [11] observed that prolonged melatonin use extending up to approximately 10 years was generally well tolerated, with most participants maintaining normal sleep patterns after discontinuation and without evidence of major developmental concerns. Nevertheless, these studies were limited by relatively small sample sizes, observational follow-up designs, reliance on self-reported measures, and the absence of detailed endocrine or hormonal evaluations. Furthermore, objective assessments of reproductive hormones, neurocognitive performance, and long-term psychological outcomes were not consistently performed. Additionally, van Geijlswijk et al. [6] and Zwart et al. [11] underscored the necessity of further research into melatonin’s influence on mental health, reproductive development, and potential interactions with other medications. Given melatonin’s physiological role in circadian rhythm regulation and its potential interaction with the hypothalamic–pituitary–gonadal axis, concerns regarding delayed pubertal maturation and endocrine alterations remain clinically relevant. Therefore, although current evidence suggests a generally favorable long-term safety profile, the available data are still insufficient to definitively establish the safety of chronic melatonin use in children. Our review also aligns with van Maanen et al. [12], who recommended future studies comparing melatonin with alternative long-term treatment approaches, including light therapy and behavioral interventions, to better determine the most effective management strategy for pediatric SOI. Future large-scale prospective studies with standardized monitoring of pubertal maturation, endocrine markers, cognitive development, and mental health outcomes are required to better define the long-term risk-benefit profile of melatonin therapy in children.

In order to obtain more precise data regarding the effects of melatonin, future research should prioritize large-scale, multi-center trials involving diverse pediatric populations and incorporating objective sleep assessments such as actigraphy and polysomnography. Additional studies are also needed to establish individualized dosing strategies, optimize circadian timing of administration, and compare melatonin with behavioral and light-based interventions. Although our findings support the therapeutic value of melatonin for pediatric SOI, further investigation remains necessary to clarify its optimal long-term use and safety.

## Conclusion

This systematic review highlights that melatonin appears to be an effective and well-tolerated option for managing sleep onset insomnia in children aged 6 to 12 years. It showed improvements in key sleep parameters, including reduced sleep latency and earlier sleep onset. Despite the promising outcomes, the current evidence is limited by variability among studies and a lack of long-term data. Therefore, further high-quality research is needed to establish clear guidelines for its safe and optimal use in pediatric care.

## Supplementary Information

Supplementary Material 1.

Supplementary Material 2.

Supplementary Material 3.

## Data Availability

No datasets were generated or analysed during the current study.
