# Supplementary material for: Evaluating the efficacy and safety of melatonin for sleep onset insomnia in children aged 6–12: a systematic review and meta-analysis
Source: BMC Pediatr. 2026 Aug 15;26:1159. doi: 10.1186/s12887-026-07505-y (PMC13753165; doi:10.1186/s12887-026-07505-y)
Supplement: Supplementary file 1 [file 12887_2026_7505_MOESM1_ESM.pdf]

Table 1: Summary and Characteristics of the Included Studies.

Last Name of Author	Year	Journal	Study Design	Country	Total Number of Patients	Inclusion Criteria (Summary)	Exclusion Criteria (Summary)
Heijden	2005	J Sleep Research	Randomized, placebo-controlled, double-blind trial	Netherlands	110	Children 6–12 years with idiopathic chronic sleep onset insomnia, sleep latency > 30 min	Sleep maintenance insomnia, disturbed sleep architecture, intellectual disability , prior melatonin use, certain medications
Hoebert	2009	J Pineal Research	Randomized, double-blind study	Netherlands	105	Children 6–12 years with ADHD, IQ > 80, chronic sleep onset insomnia	Not mentioned (NM)
Jain	2015	Sleep Medicine	Randomized, double-blind crossover study	USA (Cincinnati)	11	Children 6–11 years, pre-pubertal, epilepsy, normal development, sleep issues	Obstructive sleep apnea, PLM disorder, psychiatric disorders, prior melatonin use, certain medications
Maanen	2017	Sleep Research Society (Sleep)	Randomized controlled study	Netherlands	84	Children 7–12 years with chronic sleep onset problems >1 month, sleep latency >30 min	Psychiatric disorders (except ADHD/autism), chronic pain, epilepsy, prior melatonin use, certain medications
Smits	2001	J Child Neurology	Randomized, double-blind, placebo-controlled trial	Netherlands	40	Children 6–12 years with chronic sleep onset insomnia >1 year, sleep latency >30 min	Disturbed sleep architecture, sleep maintenance insomnia, intellectual disability , prior

							melatonin use, certain medications
Smits	2003	J Am Acad Child Adolesc Psychiatry	Randomized, double-blind, placebo-controlled trial	Netherlands	62	Children 6–12 years, good health, idiopathic chronic sleep onset insomnia >1 year	Disturbed sleep architecture, sleep maintenance insomnia, intellectual disability , prior melatonin use, certain medications
Van Geijlswijk	2010	Springer	Randomized, placebo-controlled, double-blind trial	Netherlands	72	Children 6–12 years with chronic sleep onset insomnia >1 year, sleep latency >30 min	Psychiatric/pedagogic SOI, intellectual disability, epilepsy, chronic pain, prior melatonin use, certain medications
Van Geijlswijk	2011	Springer	Cohort study	Netherlands	59	Children 6–12 years with chronic idiopathic sleep onset insomnia >1 year, good health	Psychiatric/pedagogic SOI, intellectual disability, epilepsy, chronic pain, prior melatonin use, certain medications
Heijden	2007	J Am Acad Child Adolesc Psychiatry	Randomized, double-blind, placebo-controlled study	Netherlands	107*	Children 6–12 years with ADHD and chronic sleep onset insomnia, IQ > 80	Total IQ < 80, pervasive developmental disorder, epilepsy, chronic pain, prior melatonin use, certain medications
Zwart	2018	Healthcare	Cohort study	Netherlands	69	Children 6–12 years with chronic sleep onset insomnia >1 year, non-responsive to sleep hygiene	Psychiatric/pedagogic SOI, intellectual disability, epilepsy, chronic pain, prior melatonin use, certain medications

*Note: For Heijden (2007), 107 patients were enrolled, with 105 completing the study.

Table 2. Melatonin Treatment Details Across Studies.

Last Name of Author	Melatonin Dosage	Dosage Form	Administration Time	Duration of Treatment
Heijden	5 mg	Not mentioned (NM)	18:00 or 19:00 h	4 weeks
Heijden	3 mg (<40 kg); 6 mg (>40 kg)	Fast-release tablets	7:00 h	4 weeks
Hoebert	0.5–10 mg (mean: 4.23 ± 0.27 mg)	Not mentioned (NM)	18:30–23:00 (mean: 19:53)	1–57 months (mean: 18.28 ± 3.0)
Jain	9 mg	Sustained-release melatonin	30 min prior to bedtime	4 weeks
Maanen	3 mg	Tablets	19:00 h	3–4 weeks
Smits	5 mg	Fast-release tablet	6:00 h	4 weeks + 1-week washout + 4-week crossover
Smits	5 mg	Fast-release tablet mixed with carboxymethylcellulose	7:00 h	4 weeks
Van Geijlswijk	0.05–0.15 mg/kg (by group)	Capsules	17:58–20:42 h	6 nights
Van Geijlswijk	Mean: 2.7 mg (0.3–10 mg range)	Not applicable (N/A)	Not applicable (N/A)	3.1 years
Zwart	0.5–5.0 mg	Not mentioned (NM)	19:00–23:00 h	9.6–11.9 years

Table 3. Key Findings, Limitations, Study Gaps, and Recommendations Across Studies on Melatonin for Sleep Onset Insomnia

Last Name of Author	Main Findings	Limitations	Study Gaps	Recommendations
Heijden	Children with idiopathic chronic SOI: Early evening melatonin treatment advanced sleep onset and endogenous onset, more	Actigraphy/saliva sample data loss, fixed melatonin timing ignored individual circadian	Limited exploration of melatonin's soporific vs. chronobiotic effects, lack of long-term	Use melatonin cautiously after non-pharmacological treatments fail, investigate long-term

	effective with delayed pretreatment onset.	variability, small sample size reduction.	data, no comparison with other treatments, limited generalizability.	effects and developmental impacts.
Heijden	Melatonin advanced sleep onset (~1 hour) and total sleep time (41 min) in ADHD/SOI children, no effect on behavior, cognition, or quality of life, no significant adverse events.	2-year follow-up may miss long-term effects, excludes stimulant-treated ADHD children, missing behavior/quality-of-life data, actigraphy limits sleep quality assessment.	No investigation of melatonin's effects on specific ADHD behaviors, sleep quality, or external factors; lacks long-term data on diverse populations.	Explore long-term melatonin effects on sleep, behavior, and cognition in diverse ADHD populations, including stimulant users; use polysomnography for sleep quality.
Hoebert	Melatonin effectively treated sleep onset insomnia in ADHD children long-term but did not result in a cure; discontinuation led to relapse, resuming treatment common.	Lack of systematic co-medication data, no measures for long-term pubertal/fertility effects, retrospective design, no control group.	Limited data on adverse events timing, impact of comorbidities (e.g., anxiety, depression), and pubertal/fertility effects.	Research melatonin's impact on pubertal development, efficacy across neurodevelopmental disorders, and measure pretreatment DLMO.
Jain	Sustained-release melatonin reduced sleep latency (p=0.02) and waking after sleep onset (p=0.04) in epileptic children, improved N3 sleep, prolonged REM latency, no increased seizures.	Small sample size, excluded refractory seizures, data loss in two subjects affected secondary outcomes, no significant actigraphy/subjective sleep changes.	Limited understanding of melatonin's role in refractory epilepsy, insufficient power for secondary outcomes, need for EEG/long-term effect research.	Conduct larger studies on melatonin's efficacy, safety, and tolerance in epilepsy, including diverse epilepsy types and sleep disturbances.
Maanen	Melatonin treatment was more effective than light therapy for SOI with delayed melatonin onset in children, making it the preferred clinical option.	Fixed light therapy timing, no placebo for light therapy, uncontrolled evening light exposure, unassessed sleep hygiene impact, short	Limited research on light therapy for pediatric SOI, optimal dosage/timing for both treatments, long-term impacts, specific groups (e.g.,	Research different light therapy protocols, conduct long-term studies on melatonin/light therapy effects, target specific groups, explore sleep hygiene

		duration, lack of DLMO norms.	psychiatric disorders), sleep hygiene effects.	integration, optimize dosage/timing.
Smits	Melatonin advanced sleep onset (57 min), reduced sleep latency (17 min), advanced melatonin onset (82 min) in idiopathic SOI, improved health/sleep, modest placebo effect from parental attention.	Dutch-specific population, potential ADHD bias, no objective sleep measures (e.g., actigraphy), short follow-up.	Lack of long-term melatonin effects, limited generalizability, no comparison with other treatments, unclear optimal dose.	Investigate long-term safety/efficacy in diverse populations, limit melatonin to severe resistant insomnia with delayed onset, conduct multi-center trials.
Smits	Melatonin significantly advanced sleep onset (~1 hour) and dim light melatonin onset (57 min), increased lights-off time (34 min), total sleep time (41 min), no change in attention/wake-up time.	Small sample (40 children), short treatment (4 weeks), limited long-term safety data, uncertainty in optimal dosage/timing.	Focus on short-term effects, uncertainty in optimal dosage/timing, mechanism of action, comparison with other sleep therapies, generalizability to broader populations.	Determine optimal dosage/timing, assess long-term safety/efficacy and reproductive effects, use lowest effective dose, evaluate annually for discontinuation.
Van Geijlswijk	Melatonin (0.05 mg/kg) effectively treated CSOI, advanced sleep onset (1 hour), dim light melatonin onset (1 hour), reduced sleep latency (35 min), no dose-response (0.05–0.15 mg/kg).	Small sample (16–19/group), wide DLMO-TOA intervals, naturally determined TOA.	No dose-response above 0.05 mg/kg, need for long-term safety, individual bioavailability studies.	Evaluate dosage-TOA relationship with narrower DLMO-TOA, report all p-values, conduct long-term studies on adverse events and chronic use.
Van Geijlswijk	Melatonin use persisted in 81% of children after 3.1 years, 6 of 11 discontinuers resolved sleep issues, no rebound effects, low adverse events (e.g., 58% headaches, normal population rate), normal social development, poorer sleep vs. controls, no	Small sample (57 for long-term), single clinic, non-representative of healthy Dutch children, invalidated CSHQ for older children, short follow-up.	No dose-response analysis, Dutch-only population, limited long-term data (>3.1 years), no comparison with other interventions, no drug interactions.	Monitor long-term melatonin safety (puberty, sleep, mental health), conduct studies beyond 3 years, tailor dosing, track side effects.

	pubertal changes, high user satisfaction.			
Zwart	Long-term melatonin therapy (7.1 years) was safe, 75% of CSOI children had normal sleep without medication after 10 years.	Low statistical power, recall bias, self-reported sleep timing (no actigraphy/ polysomnography), potential overestimation of melatonin continuation, student controls may not represent general population.	Need for objective sleep measures, diverse demographics, factors in therapy cessation.	Assess long-term effects on development (physical, psychological, social) into adulthood, use diverse populations, explore chronotype-daily functioning links, investigate biological/environmental factors.
