# Supplementary material for: Evaluating the efficacy and safety of melatonin for sleep onset insomnia in children aged 6–12: a systematic review and meta-analysis
Source: BMC Pediatr. 2026 Aug 15;26:1159. doi: 10.1186/s12887-026-07505-y (PMC13753165; doi:10.1186/s12887-026-07505-y)
Supplement: Supplementary file 3 [file 12887_2026_7505_MOESM3_ESM.docx]

**Search Strategies**

The following search strategies were developed using a combination of MeSH (Medical Subject Headings) terms, Boolean operators (AND, OR), and free-text keywords tailored to the syntax of each database. No publication date restrictions were applied. Where the database allowed, the search was limited to children (ages 6-12). No language restrictions were applied.

(Melatonin OR N-acetyl-5-methoxytryptamine) AND (Child* OR Pediatric OR School-age) AND (Insomnia OR Sleep onset insomnia OR Sleep disorder OR Dyssomnia) AND (Efficacy OR Safety OR Outcome) AND (Randomized controlled trial OR Randomized clinical trial OR Clinical Trial)

| Database | Search Strategy |
| --- | --- |
| PubMed | "melatonin"[MeSH Terms] OR "melatonin"[Title/Abstract]  AND  "child"[MeSH Terms] OR "children"[Title/Abstract] OR "pediatric"[Title/Abstract]  AND  ("sleep initiation disorders"[MeSH Terms] OR "sleep onset insomnia"[Title/Abstract] OR "sleep onset"[Title/Abstract] OR "insomnia"[Title/Abstract] OR "chronic sleep disorders"[Title/Abstract] OR "sleep disorders"[Title/Abstract]) |
| OVID (MEDLINE) | exp melatonin/ or melatonin.tw.  AND  exp child/ or children.tw. or pediatric.tw.  AND  exp sleep initiation disorders/ or (sleep onset insomnia or sleep onset or insomnia).tw. or (chronic sleep disorders or sleep disorders).tw. |
| Web of Science | TS=(melatonin)  AND  TS=(children OR pediatric)  AND  TS=("sleep onset insomnia" OR "sleep onset" OR insomnia OR "chronic sleep disorders" OR "sleep disorders") |
| Google Scholar | melatonin children "sleep onset insomnia" OR "chronic sleep disorders" |

The four core search concepts across all databases were: **(1)** melatonin, **(2)** children, **(3)** sleep onset insomnia, and **(4)**chronic sleep disorders. No date restrictions were applied; however, all included studies were published between 2001 and 2018.
